# Diaqua­(1,4,8,11-tetra­aza­cyclo­tetra­decane-κ^4^ *N* ^1^,*N* ^4^,*N* ^8^,*N* ^11^)copper(II) bis­(4-methyl­benzoate) monohydrate

**DOI:** 10.1107/S1600536810026012

**Published:** 2010-07-07

**Authors:** Nur Syamimi Ahmad Tajidi, Norbani Abdullah, Zainudin Arifin, Kong Wai Tan, Seik Weng Ng

**Affiliations:** a Department of Chemistry, University of Malaya, 50603 Kuala Lumpur, Malaysia

## Abstract

The Cu^II^ atom in the title salt, [Cu(C_10_H_24_N_4_)(H_2_O)_2_](C_8_H_7_O_2_)_2_·H_2_O, is chelated by the four N atoms of the 1,4,8,11-tetra­aza­cyclo­tetra­decane (cyclam) ligand and is coordinated by two water mol­ecules in a Jahn–Teller-type of tetra­gonally distorted octa­hedral geometry. The cations, anions and lattice water mol­ecules are linked by N—H⋯O and O—H⋯O hydrogen bonds to form a layer structure parallel to (001).

## Related literature

For related (1,4,8,11-tetra­aza­cyclo­tetra­deca­ne)copper carboxyl­ates, see: Lindoy *et al.* (2003 ▶); Hunter *et al.* (2005 ▶).
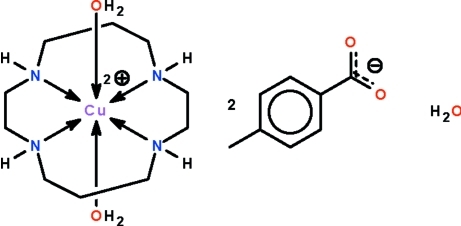


## Experimental

### 

#### Crystal data

[Cu(C_10_H_24_N_4_)(H_2_O)_2_](C_8_H_7_O_2_)_2_·H_2_O
*M* *_r_* = 588.19
Monoclinic, 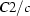

*a* = 31.925 (3) Å
*b* = 7.1779 (6) Å
*c* = 28.750 (3) Å
β = 121.880 (1)°
*V* = 5594.4 (8) Å^3^
*Z* = 8
Mo *K*α radiation
μ = 0.83 mm^−1^
*T* = 100 K
0.30 × 0.10 × 0.05 mm

#### Data collection

Bruker SMART APEX diffractometer
Absorption correction: multi-scan (*SADABS*; Sheldrick, 1996 ▶) *T*_min_ = 0.789, *T*_max_ = 0.960
26024 measured reflections
6434 independent reflections
4523 reflections with *I* > 2σ(*I*)
*R*_int_ = 0.073

#### Refinement

*R*[*F* ^2^ > 2σ(*F* ^2^)] = 0.042
*wR*(*F* ^2^) = 0.109
*S* = 1.01
6434 reflections
385 parameters
10 restraints
H atoms treated by a mixture of independent and constrained refinement
Δρ_max_ = 0.37 e Å^−3^
Δρ_min_ = −0.53 e Å^−3^

### 

Data collection: *APEX2* (Bruker, 2009 ▶); cell refinement: *SAINT* (Bruker, 2009 ▶); data reduction: *SAINT*; program(s) used to solve structure: *SHELXS97* (Sheldrick, 2008 ▶); program(s) used to refine structure: *SHELXL97* (Sheldrick, 2008 ▶); molecular graphics: *X-SEED* (Barbour, 2001 ▶); software used to prepare material for publication: *publCIF* (Westrip, 2010 ▶).

## Supplementary Material

Crystal structure: contains datablocks global, I. DOI: 10.1107/S1600536810026012/bt5288sup1.cif

Structure factors: contains datablocks I. DOI: 10.1107/S1600536810026012/bt5288Isup2.hkl

Additional supplementary materials:  crystallographic information; 3D view; checkCIF report

## Figures and Tables

**Table 1:** Selected bond lengths (Å)

Cu1—N1	2.025 (2)
Cu1—N2	2.012 (2)
Cu1—N3	2.028 (2)
Cu1—N4	2.010 (2)
Cu1—O1w	2.481 (2)
Cu1—O2w	2.531 (2)

**Table 2:** Hydrogen-bond geometry (Å, °)

*D*—H⋯*A*	*D*—H	H⋯*A*	*D*⋯*A*	*D*—H⋯*A*
N1—H1⋯O2	0.85 (1)	2.13 (1)	2.971 (3)	167 (3)
N2—H2⋯O1^i^	0.86 (1)	2.06 (2)	2.847 (3)	151 (3)
N3—H3⋯O3w^i^	0.86 (1)	2.57 (2)	3.291 (3)	143 (2)
N4—H4⋯O3^ii^	0.86 (1)	2.14 (2)	2.927 (3)	152 (3)
O1w—H11⋯O1	0.84 (1)	1.95 (1)	2.792 (2)	177 (4)
O1w—H12⋯O3w	0.84 (1)	1.96 (1)	2.795 (3)	173 (3)
O2w—H21⋯O3	0.84 (1)	1.99 (1)	2.825 (3)	173 (3)
O2w—H22⋯O2^i^	0.84 (1)	1.98 (1)	2.813 (3)	172 (3)
O3w—H31⋯O4^iii^	0.83 (1)	2.02 (1)	2.835 (3)	166 (4)
OwW—H32⋯O4^ii^	0.84 (1)	1.85 (1)	2.688 (3)	174 (3)

Symmetry codes: (i) 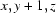; (ii) 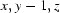; (iii) 
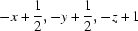
.

**Fig. 1.**
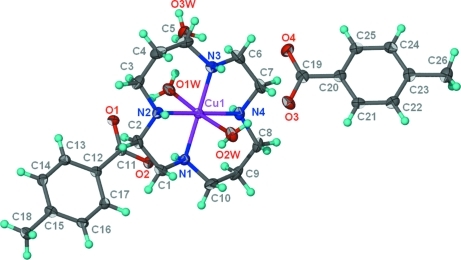
Anisotropic displacement ellipsoid plot (Barbour, 2001) of [Cu(H2O)2(C10H24N4)]2+ 2(CH3C6H4CO2)-.H2O at the 70% probability level; hydrogen atoms are drawn as spheres of arbitrary radius.

## supplementary crystallographic information

### Comment

The copper(II) ion forms a number of complexes with 1,4,8,11-tetraazacyclotetradecane in which the metal atom is coordinated by the four amino donor-atoms of the cyclic ligand. Among the carboxylate derivatives, neither the acetate nor the benzoate ions bind directly to the copper atom. The copper atom is coordinated instead by water molecules so that the carboxylate group interacts indirectly with the metal atom through the coordinated water molecules (Hunter *et al.*, 2005; Lindoy *et al.*, 2003). The copper(II) atom in the salt, [Cu(H_2_O)_2_(C_10_H_24_N_4_)]^2+^ 2(CH_3_C_6_H_4_CO_2_)^-.^H_2_O (Scheme I), is chelated by the four nitrogen atoms of the cyclam ligand and is coordinated by two water molecules in a Jahn-Teller type of tetragonally distorted octahedral geometry (Fig. 1). The cations, anions and lattice water molecules are linked by N–H···O and O–H···O hydrogen bonds to form a layer structure.

### Experimental

1,4,8,11-Tetraazacyclotetradecane (0.50 g, 2.50 mmol) dissolved in ethanol (25 ml) was mixed with a suspension of copper *p*-toluate (0.68 g, 2.5 mmol) in ethanol (50 ml) to give a purple solution. The solution was heated for an hour and then filtered. Prismatic crystals separated from the solution when it was left to cool slowly.

### Refinement

Carbon-bound H-atoms were placed in calculated positions (C—H 0.95 to 0.98 Å) and were included in the refinement in the riding model approximation, with *U*(H) set to 1.2 to 1.5*U*(C).

The amino and water H-atoms were located in a difference Fourier map, and were refined isotropically with distance restraints of N–H 0.86±0.01, O–H 0.84±0.01 Å.

### Crystal data

**Table:**

[Cu(C_10_H_24_N_4_)(H_2_O)_2_](C_8_H_7_O_2_)_2_·H_2_O	*F*(000) = 2504
*M**_r_* = 588.19	*D*_x_ = 1.397 Mg m^−^^3^
Monoclinic, *C*2/*c*	Mo *K*α radiation, λ = 0.71073 Å
Hall symbol: -C 2yc	Cell parameters from 3327 reflections
*a* = 31.925 (3) Å	θ = 2.4–28.3°
*b* = 7.1779 (6) Å	µ = 0.83 mm^−^^1^
*c* = 28.750 (3) Å	*T* = 100 K
β = 121.880 (1)°	Block, purple
*V* = 5594.4 (8) Å^3^	0.30 × 0.10 × 0.05 mm
*Z* = 8	

### Data collection

**Table:**

Bruker SMART APEX diffractometer	6434 independent reflections
Radiation source: fine-focus sealed tube	4523 reflections with *I* > 2σ(*I*)
graphite	*R*_int_ = 0.073
ω scans	θ_max_ = 27.5°, θ_min_ = 1.6°
Absorption correction: multi-scan (*SADABS*; Sheldrick, 1996)	*h* = −39→41
*T*_min_ = 0.789, *T*_max_ = 0.960	*k* = −8→9
26024 measured reflections	*l* = −37→37

### Refinement

**Table:**

Refinement on *F*^2^	Primary atom site location: structure-invariant direct methods
Least-squares matrix: full	Secondary atom site location: difference Fourier map
*R*[*F*^2^ > 2σ(*F*^2^)] = 0.042	Hydrogen site location: inferred from neighbouring sites
*wR*(*F*^2^) = 0.109	H atoms treated by a mixture of independent and constrained refinement
*S* = 1.01	*w* = 1/[σ^2^(*F*_o_^2^) + (0.0467*P*)^2^ + 3.0196*P*] where *P* = (*F*_o_^2^ + 2*F*_c_^2^)/3
6434 reflections	(Δ/σ)_max_ = 0.002
385 parameters	Δρ_max_ = 0.37 e Å^−^^3^
10 restraints	Δρ_min_ = −0.53 e Å^−^^3^

### Special details

**Table:**

Geometry. All e.s.d.'s (except the e.s.d. in the dihedral angle between two l.s. planes) are estimated using the full covariance matrix. The cell e.s.d.'s are taken into account individually in the estimation of e.s.d.'s in distances, angles and torsion angles; correlations between e.s.d.'s in cell parameters are only used when they are defined by crystal symmetry. An approximate (isotropic) treatment of cell e.s.d.'s is used for estimating e.s.d.'s involving l.s. planes.

### Fractional atomic coordinates and isotropic or equivalent isotropic displacement parameters (Å^2^)

**Table:**

	*x*	*y*	*z*	*U*_iso_*/*U*_eq_	
Cu1	0.412658 (11)	0.34713 (4)	0.559593 (12)	0.01501 (10)	
O1	0.47818 (6)	−0.1771 (2)	0.66496 (7)	0.0174 (4)	
O2	0.50203 (6)	−0.1284 (2)	0.60553 (7)	0.0182 (4)	
O3	0.33915 (6)	0.8743 (2)	0.44684 (7)	0.0221 (4)	
O1W	0.39877 (7)	0.0374 (3)	0.58743 (8)	0.0204 (4)	
O2W	0.42053 (7)	0.6641 (3)	0.52635 (8)	0.0220 (4)	
O3W	0.30940 (8)	−0.1475 (3)	0.54990 (8)	0.0291 (5)	
O4	0.27168 (7)	0.7719 (3)	0.44369 (7)	0.0249 (4)	
N1	0.47989 (8)	0.2709 (3)	0.57497 (8)	0.0159 (5)	
H1	0.4817 (10)	0.1556 (16)	0.5829 (11)	0.016 (7)*	
N2	0.44938 (8)	0.4440 (3)	0.63695 (8)	0.0164 (5)	
H2	0.4495 (10)	0.5631 (15)	0.6338 (11)	0.024 (8)*	
N3	0.34589 (8)	0.4280 (3)	0.54508 (9)	0.0185 (5)	
H3	0.3441 (10)	0.5449 (16)	0.5386 (11)	0.020 (8)*	
N4	0.37563 (8)	0.2541 (3)	0.48193 (8)	0.0174 (5)	
H4	0.3719 (11)	0.1360 (16)	0.4836 (12)	0.031 (9)*	
C1	0.51636 (9)	0.3789 (4)	0.62349 (10)	0.0187 (6)	
H1A	0.5177	0.5089	0.6128	0.022*	
H1B	0.5495	0.3229	0.6397	0.022*	
C2	0.50050 (9)	0.3757 (4)	0.66449 (10)	0.0204 (6)	
H2A	0.5025	0.2472	0.6780	0.024*	
H2B	0.5224	0.4564	0.6961	0.024*	
C3	0.42706 (10)	0.4097 (4)	0.66988 (11)	0.0228 (6)	
H3A	0.4482	0.4658	0.7066	0.027*	
H3B	0.4256	0.2737	0.6747	0.027*	
C4	0.37544 (10)	0.4909 (4)	0.64307 (12)	0.0272 (7)	
H4A	0.3645	0.4832	0.6695	0.033*	
H4B	0.3770	0.6245	0.6356	0.033*	
C5	0.33683 (10)	0.3979 (4)	0.59015 (12)	0.0248 (6)	
H5A	0.3367	0.2625	0.5965	0.030*	
H5B	0.3039	0.4478	0.5790	0.030*	
C6	0.30837 (9)	0.3366 (4)	0.49338 (11)	0.0231 (6)	
H6A	0.2765	0.4031	0.4773	0.028*	
H6B	0.3034	0.2062	0.5006	0.028*	
C7	0.32624 (9)	0.3411 (4)	0.45436 (10)	0.0222 (6)	
H7A	0.3032	0.2717	0.4205	0.027*	
H7B	0.3281	0.4713	0.4443	0.027*	
C8	0.39989 (10)	0.2750 (4)	0.45028 (10)	0.0218 (6)	
H8A	0.4030	0.4093	0.4447	0.026*	
H8B	0.3789	0.2173	0.4138	0.026*	
C9	0.45044 (11)	0.1862 (4)	0.47853 (11)	0.0248 (6)	
H9A	0.4618	0.1804	0.4525	0.030*	
H9B	0.4480	0.0569	0.4888	0.030*	
C10	0.48843 (10)	0.2908 (4)	0.52945 (11)	0.0232 (6)	
H10A	0.5217	0.2432	0.5413	0.028*	
H10B	0.4874	0.4245	0.5205	0.028*	
C11	0.50985 (9)	−0.1325 (3)	0.65344 (10)	0.0150 (5)	
C12	0.56003 (9)	−0.0723 (4)	0.70001 (10)	0.0146 (5)	
C13	0.57185 (9)	−0.0785 (4)	0.75417 (10)	0.0166 (5)	
H13	0.5486	−0.1259	0.7623	0.020*	
C14	0.61733 (9)	−0.0159 (4)	0.79610 (10)	0.0164 (5)	
H14	0.6253	−0.0247	0.8329	0.020*	
C15	0.65166 (9)	0.0597 (4)	0.78562 (10)	0.0170 (5)	
C16	0.63953 (9)	0.0675 (4)	0.73143 (10)	0.0173 (5)	
H16	0.6623	0.1198	0.7233	0.021*	
C17	0.59464 (9)	0.0000 (4)	0.68914 (10)	0.0166 (5)	
H17	0.5874	0.0030	0.6526	0.020*	
C18	0.70144 (9)	0.1270 (4)	0.83133 (10)	0.0207 (6)	
H18A	0.7272	0.0788	0.8258	0.031*	
H18B	0.7073	0.0823	0.8665	0.031*	
H18C	0.7021	0.2635	0.8314	0.031*	
C19	0.29372 (10)	0.8409 (4)	0.42210 (10)	0.0194 (6)	
C20	0.26273 (9)	0.8875 (3)	0.36134 (11)	0.0177 (6)	
C21	0.28361 (9)	0.9741 (4)	0.33499 (10)	0.0182 (6)	
H21A	0.3177	1.0048	0.3551	0.022*	
C22	0.25492 (10)	1.0159 (4)	0.27958 (11)	0.0210 (6)	
H22A	0.2695	1.0782	0.2624	0.025*	
C23	0.20511 (10)	0.9682 (4)	0.24858 (10)	0.0193 (6)	
C24	0.18443 (10)	0.8818 (4)	0.27541 (11)	0.0197 (6)	
H24	0.1505	0.8489	0.2554	0.024*	
C25	0.21318 (9)	0.8438 (4)	0.33112 (10)	0.0183 (5)	
H25	0.1984	0.7865	0.3488	0.022*	
C26	0.17495 (11)	1.0007 (4)	0.18760 (11)	0.0268 (7)	
H26A	0.1399	1.0042	0.1752	0.040*	
H26B	0.1809	0.8995	0.1689	0.040*	
H26C	0.1845	1.1197	0.1791	0.040*	
H11	0.4219 (9)	−0.029 (4)	0.6111 (11)	0.052 (11)*	
H12	0.3716 (7)	−0.018 (5)	0.5734 (13)	0.057 (12)*	
H21	0.3952 (7)	0.724 (4)	0.5043 (10)	0.036 (10)*	
H22	0.4431 (9)	0.736 (4)	0.5482 (11)	0.036 (10)*	
H31	0.2885 (11)	−0.178 (5)	0.5575 (15)	0.062 (13)*	
H32	0.2992 (11)	−0.168 (5)	0.5169 (5)	0.038 (10)*	

### Atomic displacement parameters (Å^2^)

**Table:**

	*U*^11^	*U*^22^	*U*^33^	*U*^12^	*U*^13^	*U*^23^
Cu1	0.01367 (16)	0.01757 (17)	0.01320 (15)	0.00050 (13)	0.00669 (12)	−0.00049 (13)
O1	0.0142 (9)	0.0203 (10)	0.0183 (9)	−0.0012 (7)	0.0089 (8)	0.0002 (7)
O2	0.0184 (9)	0.0215 (10)	0.0138 (8)	−0.0010 (8)	0.0078 (7)	−0.0007 (7)
O3	0.0153 (9)	0.0199 (10)	0.0230 (10)	−0.0016 (8)	0.0047 (8)	0.0001 (8)
O1W	0.0165 (10)	0.0175 (10)	0.0236 (10)	0.0018 (8)	0.0081 (9)	0.0044 (8)
O2W	0.0203 (10)	0.0178 (10)	0.0195 (10)	−0.0016 (9)	0.0048 (9)	−0.0017 (8)
O3W	0.0240 (11)	0.0426 (13)	0.0185 (10)	−0.0106 (10)	0.0098 (9)	0.0009 (10)
O4	0.0256 (10)	0.0320 (12)	0.0197 (9)	−0.0064 (9)	0.0139 (9)	−0.0013 (8)
N1	0.0176 (11)	0.0144 (12)	0.0166 (11)	0.0013 (9)	0.0097 (9)	0.0025 (9)
N2	0.0186 (11)	0.0147 (12)	0.0171 (11)	0.0002 (9)	0.0102 (9)	0.0011 (9)
N3	0.0183 (11)	0.0150 (12)	0.0233 (12)	0.0010 (9)	0.0117 (10)	0.0010 (9)
N4	0.0198 (11)	0.0130 (12)	0.0158 (11)	−0.0008 (9)	0.0069 (9)	0.0015 (9)
C1	0.0145 (12)	0.0175 (14)	0.0219 (13)	−0.0004 (10)	0.0080 (11)	−0.0005 (10)
C2	0.0177 (13)	0.0223 (15)	0.0148 (12)	0.0025 (11)	0.0043 (11)	0.0005 (10)
C3	0.0306 (15)	0.0239 (15)	0.0188 (13)	−0.0005 (12)	0.0165 (12)	−0.0014 (11)
C4	0.0317 (16)	0.0285 (17)	0.0326 (16)	−0.0015 (13)	0.0246 (14)	−0.0052 (13)
C5	0.0255 (15)	0.0229 (15)	0.0347 (16)	−0.0007 (12)	0.0219 (13)	−0.0012 (12)
C6	0.0148 (13)	0.0184 (14)	0.0266 (14)	0.0008 (11)	0.0044 (11)	0.0027 (12)
C7	0.0188 (13)	0.0207 (14)	0.0189 (13)	0.0020 (12)	0.0043 (11)	0.0030 (11)
C8	0.0288 (15)	0.0230 (15)	0.0140 (12)	−0.0003 (12)	0.0115 (12)	0.0010 (11)
C9	0.0373 (17)	0.0242 (16)	0.0240 (14)	0.0029 (13)	0.0238 (13)	0.0030 (12)
C10	0.0249 (14)	0.0270 (16)	0.0256 (14)	−0.0005 (12)	0.0188 (12)	0.0017 (12)
C11	0.0164 (12)	0.0108 (12)	0.0171 (12)	0.0036 (10)	0.0085 (10)	0.0013 (10)
C12	0.0154 (12)	0.0117 (12)	0.0171 (12)	0.0015 (10)	0.0087 (10)	0.0002 (10)
C13	0.0156 (12)	0.0159 (13)	0.0193 (13)	0.0011 (10)	0.0100 (11)	0.0015 (10)
C14	0.0189 (13)	0.0161 (13)	0.0138 (12)	0.0008 (10)	0.0084 (10)	−0.0021 (10)
C15	0.0161 (12)	0.0120 (13)	0.0216 (13)	0.0013 (10)	0.0091 (11)	−0.0026 (10)
C16	0.0135 (12)	0.0173 (13)	0.0212 (13)	0.0003 (10)	0.0093 (11)	0.0029 (11)
C17	0.0169 (13)	0.0169 (13)	0.0164 (12)	0.0021 (10)	0.0090 (10)	0.0035 (10)
C18	0.0165 (13)	0.0221 (15)	0.0202 (13)	−0.0012 (11)	0.0073 (11)	−0.0010 (11)
C19	0.0217 (13)	0.0153 (13)	0.0214 (13)	0.0021 (11)	0.0115 (11)	−0.0037 (11)
C20	0.0201 (13)	0.0141 (13)	0.0208 (13)	0.0013 (10)	0.0120 (11)	−0.0017 (10)
C21	0.0180 (13)	0.0154 (14)	0.0239 (13)	−0.0015 (10)	0.0129 (11)	−0.0030 (11)
C22	0.0251 (14)	0.0182 (14)	0.0250 (14)	−0.0009 (11)	0.0168 (12)	−0.0003 (11)
C23	0.0234 (14)	0.0155 (13)	0.0185 (13)	0.0027 (11)	0.0107 (11)	−0.0029 (10)
C24	0.0170 (13)	0.0189 (14)	0.0226 (14)	−0.0014 (11)	0.0101 (11)	−0.0042 (11)
C25	0.0206 (13)	0.0140 (13)	0.0230 (13)	−0.0018 (11)	0.0134 (11)	−0.0028 (11)
C26	0.0311 (16)	0.0298 (17)	0.0204 (14)	0.0000 (13)	0.0142 (13)	0.0000 (12)

### Geometric parameters (Å, °)

**Table:**

Cu1—N1	2.025 (2)	C6—H6A	0.9900
Cu1—N2	2.012 (2)	C6—H6B	0.9900
Cu1—N3	2.028 (2)	C7—H7A	0.9900
Cu1—N4	2.010 (2)	C7—H7B	0.9900
Cu1—O1w	2.481 (2)	C8—C9	1.512 (4)
Cu1—O2w	2.531 (2)	C8—H8A	0.9900
O1—C11	1.260 (3)	C8—H8B	0.9900
O2—C11	1.264 (3)	C9—C10	1.517 (4)
O3—C19	1.256 (3)	C9—H9A	0.9900
O1W—H11	0.841 (10)	C9—H9B	0.9900
O1W—H12	0.839 (10)	C10—H10A	0.9900
O2W—H21	0.838 (10)	C10—H10B	0.9900
O2W—H22	0.837 (10)	C11—C12	1.510 (3)
O3W—H31	0.833 (10)	C12—C13	1.396 (3)
O3W—H32	0.838 (10)	C12—C17	1.397 (3)
O4—C19	1.260 (3)	C13—C14	1.384 (3)
N1—C10	1.478 (3)	C13—H13	0.9500
N1—C1	1.479 (3)	C14—C15	1.391 (4)
N1—H1	0.852 (10)	C14—H14	0.9500
N2—C2	1.472 (3)	C15—C16	1.394 (3)
N2—C3	1.476 (3)	C15—C18	1.510 (3)
N2—H2	0.860 (10)	C16—C17	1.388 (3)
N3—C6	1.481 (3)	C16—H16	0.9500
N3—C5	1.485 (3)	C17—H17	0.9500
N3—H3	0.855 (10)	C18—H18A	0.9800
N4—C7	1.478 (3)	C18—H18B	0.9800
N4—C8	1.482 (3)	C18—H18C	0.9800
N4—H4	0.861 (10)	C19—C20	1.522 (4)
C1—C2	1.509 (4)	C20—C25	1.380 (3)
C1—H1A	0.9900	C20—C21	1.392 (4)
C1—H1B	0.9900	C21—C22	1.388 (4)
C2—H2A	0.9900	C21—H21A	0.9500
C2—H2B	0.9900	C22—C23	1.394 (4)
C3—C4	1.519 (4)	C22—H22A	0.9500
C3—H3A	0.9900	C23—C24	1.398 (4)
C3—H3B	0.9900	C23—C26	1.507 (3)
C4—C5	1.516 (4)	C24—C25	1.389 (4)
C4—H4A	0.9900	C24—H24	0.9500
C4—H4B	0.9900	C25—H25	0.9500
C5—H5A	0.9900	C26—H26A	0.9800
C5—H5B	0.9900	C26—H26B	0.9800
C6—C7	1.504 (4)	C26—H26C	0.9800
			
N4—Cu1—N2	179.18 (10)	C7—C6—H6B	110.0
N4—Cu1—N1	94.96 (9)	H6A—C6—H6B	108.4
N2—Cu1—N1	85.44 (9)	N4—C7—C6	108.0 (2)
N4—Cu1—N3	85.92 (9)	N4—C7—H7A	110.1
N2—Cu1—N3	93.67 (9)	C6—C7—H7A	110.1
N1—Cu1—N3	178.90 (10)	N4—C7—H7B	110.1
N4—Cu1—O1W	87.71 (8)	C6—C7—H7B	110.1
N2—Cu1—O1W	93.00 (8)	H7A—C7—H7B	108.4
N1—Cu1—O1W	92.10 (8)	N4—C8—C9	112.5 (2)
N3—Cu1—O1W	88.59 (8)	N4—C8—H8A	109.1
N4—Cu1—O2W	89.81 (8)	C9—C8—H8A	109.1
N2—Cu1—O2W	89.46 (8)	N4—C8—H8B	109.1
N1—Cu1—O2W	91.16 (8)	C9—C8—H8B	109.1
N3—Cu1—O2W	88.18 (8)	H8A—C8—H8B	107.8
O1W—Cu1—O2W	176.06 (6)	C8—C9—C10	113.1 (2)
Cu1—O1W—H11	123 (3)	C8—C9—H9A	109.0
Cu1—O1W—H12	126 (3)	C10—C9—H9A	109.0
H11—O1W—H12	111 (4)	C8—C9—H9B	109.0
Cu1—O2W—H21	120 (2)	C10—C9—H9B	109.0
Cu1—O2W—H22	119 (2)	H9A—C9—H9B	107.8
H21—O2W—H22	111 (3)	N1—C10—C9	112.3 (2)
H31—O3W—H32	112 (3)	N1—C10—H10A	109.1
C10—N1—C1	111.3 (2)	C9—C10—H10A	109.1
C10—N1—Cu1	116.79 (16)	N1—C10—H10B	109.1
C1—N1—Cu1	106.24 (15)	C9—C10—H10B	109.1
C10—N1—H1	108.2 (19)	H10A—C10—H10B	107.9
C1—N1—H1	110.4 (18)	O1—C11—O2	124.6 (2)
Cu1—N1—H1	103.6 (19)	O1—C11—C12	117.6 (2)
C2—N2—C3	111.9 (2)	O2—C11—C12	117.8 (2)
C2—N2—Cu1	108.23 (16)	C13—C12—C17	118.8 (2)
C3—N2—Cu1	117.02 (16)	C13—C12—C11	121.0 (2)
C2—N2—H2	108.9 (19)	C17—C12—C11	120.1 (2)
C3—N2—H2	105.3 (19)	C14—C13—C12	120.2 (2)
Cu1—N2—H2	105.0 (19)	C14—C13—H13	119.9
C6—N3—C5	112.6 (2)	C12—C13—H13	119.9
C6—N3—Cu1	106.55 (16)	C13—C14—C15	121.5 (2)
C5—N3—Cu1	116.53 (17)	C13—C14—H14	119.3
C6—N3—H3	106.9 (19)	C15—C14—H14	119.3
C5—N3—H3	108.2 (19)	C14—C15—C16	118.2 (2)
Cu1—N3—H3	105.4 (19)	C14—C15—C18	121.6 (2)
C7—N4—C8	112.5 (2)	C16—C15—C18	120.2 (2)
C7—N4—Cu1	106.88 (16)	C17—C16—C15	120.9 (2)
C8—N4—Cu1	117.20 (16)	C17—C16—H16	119.5
C7—N4—H4	108 (2)	C15—C16—H16	119.5
C8—N4—H4	105 (2)	C16—C17—C12	120.4 (2)
Cu1—N4—H4	107 (2)	C16—C17—H17	119.8
N1—C1—C2	107.9 (2)	C12—C17—H17	119.8
N1—C1—H1A	110.1	C15—C18—H18A	109.5
C2—C1—H1A	110.1	C15—C18—H18B	109.5
N1—C1—H1B	110.1	H18A—C18—H18B	109.5
C2—C1—H1B	110.1	C15—C18—H18C	109.5
H1A—C1—H1B	108.4	H18A—C18—H18C	109.5
N2—C2—C1	108.0 (2)	H18B—C18—H18C	109.5
N2—C2—H2A	110.1	O3—C19—O4	125.1 (2)
C1—C2—H2A	110.1	O3—C19—C20	117.7 (2)
N2—C2—H2B	110.1	O4—C19—C20	117.3 (2)
C1—C2—H2B	110.1	C25—C20—C21	118.5 (2)
H2A—C2—H2B	108.4	C25—C20—C19	120.5 (2)
N2—C3—C4	111.9 (2)	C21—C20—C19	121.0 (2)
N2—C3—H3A	109.2	C22—C21—C20	120.4 (2)
C4—C3—H3A	109.2	C22—C21—H21A	119.8
N2—C3—H3B	109.2	C20—C21—H21A	119.8
C4—C3—H3B	109.2	C21—C22—C23	121.3 (3)
H3A—C3—H3B	107.9	C21—C22—H22A	119.4
C5—C4—C3	115.4 (2)	C23—C22—H22A	119.4
C5—C4—H4A	108.4	C22—C23—C24	117.9 (2)
C3—C4—H4A	108.4	C22—C23—C26	121.2 (2)
C5—C4—H4B	108.4	C24—C23—C26	120.9 (2)
C3—C4—H4B	108.4	C25—C24—C23	120.4 (2)
H4A—C4—H4B	107.5	C25—C24—H24	119.8
N3—C5—C4	112.5 (2)	C23—C24—H24	119.8
N3—C5—H5A	109.1	C20—C25—C24	121.4 (2)
C4—C5—H5A	109.1	C20—C25—H25	119.3
N3—C5—H5B	109.1	C24—C25—H25	119.3
C4—C5—H5B	109.1	C23—C26—H26A	109.5
H5A—C5—H5B	107.8	C23—C26—H26B	109.5
N3—C6—C7	108.3 (2)	H26A—C26—H26B	109.5
N3—C6—H6A	110.0	C23—C26—H26C	109.5
C7—C6—H6A	110.0	H26A—C26—H26C	109.5
N3—C6—H6B	110.0	H26B—C26—H26C	109.5
			
N4—Cu1—N1—C10	36.50 (19)	Cu1—N3—C5—C4	56.2 (3)
N2—Cu1—N1—C10	−142.78 (19)	C3—C4—C5—N3	−67.2 (3)
O1W—Cu1—N1—C10	124.38 (18)	C5—N3—C6—C7	−168.5 (2)
O2W—Cu1—N1—C10	−53.41 (19)	Cu1—N3—C6—C7	−39.5 (2)
N4—Cu1—N1—C1	161.28 (16)	C8—N4—C7—C6	−171.8 (2)
N2—Cu1—N1—C1	−17.99 (16)	Cu1—N4—C7—C6	−41.8 (2)
O1W—Cu1—N1—C1	−110.83 (16)	N3—C6—C7—N4	55.1 (3)
O2W—Cu1—N1—C1	71.37 (16)	C7—N4—C8—C9	179.9 (2)
N1—Cu1—N2—C2	−11.11 (17)	Cu1—N4—C8—C9	55.4 (3)
N3—Cu1—N2—C2	169.54 (17)	N4—C8—C9—C10	−70.6 (3)
O1W—Cu1—N2—C2	80.77 (17)	C1—N1—C10—C9	−178.1 (2)
O2W—Cu1—N2—C2	−102.31 (17)	Cu1—N1—C10—C9	−55.9 (3)
N1—Cu1—N2—C3	−138.62 (19)	C8—C9—C10—N1	71.1 (3)
N3—Cu1—N2—C3	42.03 (19)	O1—C11—C12—C13	4.4 (4)
O1W—Cu1—N2—C3	−46.75 (19)	O2—C11—C12—C13	−178.0 (2)
O2W—Cu1—N2—C3	130.17 (18)	O1—C11—C12—C17	−172.3 (2)
N4—Cu1—N3—C6	13.16 (17)	O2—C11—C12—C17	5.3 (4)
N2—Cu1—N3—C6	−167.56 (17)	C17—C12—C13—C14	−0.7 (4)
O1W—Cu1—N3—C6	−74.65 (17)	C11—C12—C13—C14	−177.5 (2)
O2W—Cu1—N3—C6	103.09 (17)	C12—C13—C14—C15	2.0 (4)
N4—Cu1—N3—C5	139.77 (19)	C13—C14—C15—C16	−1.3 (4)
N2—Cu1—N3—C5	−40.95 (19)	C13—C14—C15—C18	−179.6 (2)
O1W—Cu1—N3—C5	51.96 (18)	C14—C15—C16—C17	−0.7 (4)
O2W—Cu1—N3—C5	−130.29 (18)	C18—C15—C16—C17	177.6 (2)
N1—Cu1—N4—C7	−163.46 (17)	C15—C16—C17—C12	1.9 (4)
N3—Cu1—N4—C7	15.88 (17)	C13—C12—C17—C16	−1.2 (4)
O1W—Cu1—N4—C7	104.62 (16)	C11—C12—C17—C16	175.5 (2)
O2W—Cu1—N4—C7	−72.32 (16)	O3—C19—C20—C25	176.1 (2)
N1—Cu1—N4—C8	−36.14 (19)	O4—C19—C20—C25	−4.0 (4)
N3—Cu1—N4—C8	143.20 (19)	O3—C19—C20—C21	−3.7 (4)
O1W—Cu1—N4—C8	−128.05 (18)	O4—C19—C20—C21	176.2 (2)
O2W—Cu1—N4—C8	55.01 (18)	C25—C20—C21—C22	0.3 (4)
C10—N1—C1—C2	171.4 (2)	C19—C20—C21—C22	−180.0 (2)
Cu1—N1—C1—C2	43.2 (2)	C20—C21—C22—C23	−1.8 (4)
C3—N2—C2—C1	168.1 (2)	C21—C22—C23—C24	2.0 (4)
Cu1—N2—C2—C1	37.7 (2)	C21—C22—C23—C26	−175.4 (2)
N1—C1—C2—N2	−54.6 (3)	C22—C23—C24—C25	−0.7 (4)
C2—N2—C3—C4	176.4 (2)	C26—C23—C24—C25	176.7 (2)
Cu1—N2—C3—C4	−57.9 (3)	C21—C20—C25—C24	1.0 (4)
N2—C3—C4—C5	67.7 (3)	C19—C20—C25—C24	−178.7 (2)
C6—N3—C5—C4	179.8 (2)	C23—C24—C25—C20	−0.8 (4)

### Hydrogen-bond geometry (Å, °)

**Table:**

*D*—H···*A*	*D*—H	H···*A*	*D*···*A*	*D*—H···*A*
N1—H1···O2	0.85 (1)	2.13 (1)	2.971 (3)	167 (3)
N2—H2···O1^i^	0.86 (1)	2.06 (2)	2.847 (3)	151 (3)
N3—H3···O3w^i^	0.86 (1)	2.57 (2)	3.291 (3)	143 (2)
N4—H4···O3^ii^	0.86 (1)	2.14 (2)	2.927 (3)	152 (3)
O1w—H11···O1	0.84 (1)	1.95 (1)	2.792 (2)	177 (4)
O1w—H12···O3w	0.84 (1)	1.96 (1)	2.795 (3)	173 (3)
O2w—H21···O3	0.84 (1)	1.99 (1)	2.825 (3)	173 (3)
O2w—H22···O2^i^	0.84 (1)	1.98 (1)	2.813 (3)	172 (3)
O3w—H31···O4^iii^	0.83 (1)	2.02 (1)	2.835 (3)	166 (4)
OwW—H32···O4^ii^	0.84 (1)	1.85 (1)	2.688 (3)	174 (3)

Symmetry codes: (i) *x*, *y*+1, *z*; (ii) *x*, *y*−1, *z*; (iii) −*x*+1/2, −*y*+1/2, −*z*+1.
